# Adhesion and whitening efficacy of P11-4 self-assembling peptide and HAP suspension after using NaOCl as a pre-treatment agent

**DOI:** 10.1186/s12903-022-02080-x

**Published:** 2022-03-04

**Authors:** Niloofar Hojabri, Karl-Heinz Kunzelmann

**Affiliations:** Department of Conservative Dentistry and Periodontology, University Hospital, Ludwig-Maximilians-University Munich, Goethestr. 70, 80336 Munich, Germany

**Keywords:** Hydroxyapatite (HAP), Self-assembling peptide P11-4, Sodium hypochlorite (NaOCl), Bovine enamel, Color measurement, Tooth whitening

## Abstract

**Background:**

This study evaluated the adhesion and whitening efficacy of a mixture of hydroxyapatite and P11-4 self-assembling peptide (HAP-peptide) on bovine enamel after pre-treatment with low-concentrated sodium hypochlorite (NaOCl).

**Methods:**

Fifty-two caries-free bovine incisors were selected. 50 teeth were randomly allocated to five groups (n = 10). The first group was treated with a mixture of 6.25 wt% HAP and 5 ml P11-4 peptide, using NaOCl 3% as pre-treatment. Second, third and fourth groups were treated with 6.25 wt% HAP, 5 ml P11-4 peptide, and NaOCl 3%, respectively. In the fifth group, only water was applied (control group). The color of samples was measured using a spectrophotometer (USB4000-VIS-NIR-ES, Ostfildern, Germany). To evaluate color changes, ΔE values were statistically analyzed. Finally, adherence of HAP particles on two enamel surfaces with and without pre-treatment with NaOCl was analyzed with SEM.

**Results:**

It was observed that the ΔE of the HAP-peptide suspension after pre-treatment with NaOCl was significantly stronger than the control group. In contrast, the overall color changes of separate applications of HAP, peptide, and NaOCl did not differ notably from the control group. SEM observations confirmed that pre-treatment with NaOCl resulted in a more pronounced coverage of HAP on the enamel surface.

**Conclusions:**

Pre-treatment with a low-concentrated NaOCl enhanced the adherence of the HAP layer on the enamel surface, resulting in a stronger whitening effect.

**Trial registration:**

The peptide-HAP suspension is effective in improving tooth whiteness.

## Background

Dental aesthetics including tooth whiteness is of great importance for the majority of people. Therefore, tooth whitening is one of the most demanded dental treatments. [1]. In response, many tooth whitening products and methods have been made available. These include home-based products such as whitening toothpaste, gels, and bleaching trays, as well as in-office-based procedures where products containing high-concentrated bleaching agents are applied under the dentist’s supervision.

Human tooth enamel consists of calcium phosphate in the form of hydroxyapatite (HAP), Ca_5_(PO_4_)_3_(OH) [2]. Biomimetic HAP inspired by the structure and composition of natural enamel crystallites, is widely used in preventive oral health care, restoring initial cavities and removing dental plaque [3, 4]. Findings of recent studies (e.g. [5, 6]) showed that HAP as a white biomaterial can also be used in tooth whitening. In essence, the adhesion of HAP particles on the tooth surface can cause diffuse reflection, making the tooth appear brighter [7]. However, the whitening efficacy of HAP depends on the level of adhesion of its particles on the tooth surface.

Peptide self-assembly is a relatively new approach for creating synthetic super molecular architectures [12], which occurs via spontaneous and reversible noncovalent interactions between peptide chains [8, 9]. The P11-4 peptide (clinically available as Curodont Repair) with the chemical structure: Ace-Gln–Gln–Arg–Phe–Glu–Trp–Glu–Phe–Glu–Gln–Gln–NH2 responds to pH triggers and assembles in the hierarchical order of tapes, ribbons, fibrils, and fibers. This peptide is mainly used in the treatment of white spot lesions, prevention of initial caries lesions, and conducting remineralization through increasing mineral gain and inhibiting mineral loss [10, 11].

In natural enamel, HAP crystallites are glued by proteins into prisms [12]. The P11-4 is capable of forming a 3-dimensional biocompatible matrix, which mimics the natural enamel matrix proteins. This matrix can be surrounded by the calcium phosphate available in saliva, in the same way as for natural intraoral tooth remineralization. In response to specific environmental triggers, P11-4 peptide can spontaneously assemble and develop fibrillar 3D scaffolds which are capable of nucleating de novo HAP [13]. In particular, it forms ß-sheet, tape-like assemblies at a pH of lower than 7.4 [14, 15]. When pH decreases, the peptide switches from a low viscosity state to a gel state [16, 17]. The lateral chains of bioactive peptide scaffold can attract calcium ions [13].

Each fibril of the developed P11-4 network encompasses 4 ribbons. The acidic glutamate residues which are arrayed along the fibrillar surface and have a negative charge domain, can contribute to calcium-binding by supplying nucleating sites for HAP crystals. In contrast, the hydrophobic residues (e.g. Phenyl) may contribute to the assembly of the peptide [18].

Due to the aforementioned characteristics of P11-4, it can act as a bonding glue between HAP particles and the enamel surface. In other words, it increases the adherence of HAP particles to the enamel surface. Increasing the HAP thickness on the enamel surface can increase defuse reflection of light, making the tooth appear brighter in the eyes of an observer [19, 20]. However, the smear layer caused by polishing the sample’s surface after embedding and also the chromogenic particles located on the tooth’s surface may interfere with the adherence of the P11-4-HAP layer.

NaOCl is an antimicrobial and proteolytic agent. While it acts effectively in dissolving the organic matter of the smear layer, it is less effective in removing the inorganic part of the layer [21, 22]. NaOCl can act as a fat and organic solvent for vital, necrotic, and fixed tissues. Furthermore, it neutralizes amino acids and transforms them into salt and water [23]. Since a low-concentrated NaOCl with reasonably short exposure time has acceptable biological compatibility, it is widely used as an irrigation solution in root canal treatments [24, 25].

We hypothesized that the application of NaOCl 3% enhances the adhesion of HAP-peptide on the tooth surface, making the tooth appear brighter. The study aimed to evaluate the adhesion and whitening efficacy of a suspension containing 6.25 wt% HAP and 5 ml P11-4 peptide after using NaOCl 3% as pre-treatment. The null hypothesis states that the application of a mixture of peptide P11-4 and HAP suspension after pre-treatment with NaOCl 3% has no significant effect on the overall color changes (ΔE values) of enamel. Structural changes in bovine enamel surface after treatment with the HAP-peptide suspension with and without pre-treatment with NaOCl were qualitatively analyzed by SEM.

## Materials and methods

Fifty-two freshly extracted bovine incisors without roots, any stains, cracks, or cavities were randomly selected and polished using a rubber cup in a dental hand piece for 30 s with a fine prophylactic polishing paste (Proxyt, RDA 7, fine, Ivoclar Vivadent, Schaan, Liechtenstein). The teeth were provided by Vion Food Group (Vion GmbH, Waldkraiburg, Germany) The teeth were only allowed to be extracted after certain veterinary examinations ensuring that the animal was free of prions and suitable for consumption.

Fifty samples were assigned to the color measurement experiments and two samples were assigned to the SEM analysis. The samples were prepared and treated in a similar approach as [19].

### Sample preparation

Samples were embedded in resin (Technovit 4004 transparent embedding kits, Kulzer, Germany) using cuboid silicon molds (2.4 cm × 2 cm × 1.5 cm). To get a flat and standardized measurement surface, the vestibular aprismatic enamel surface of the samples was polished in order to expose an ellipse-shaped window of enamel with a major axis of approximately 10 mm and a minor axis of 6 mm. The samples were polished with 600- and 1200-grit SiC abrasive papers^1^ (Leco Corporation, St. Joseph, USA), using water cooling. They were then stored in a standard mineral water (Evian; DanoneWaters Deutschland, Frankfurt, Germany) at room temperature until the staining procedure. To stain samples, they were immersed into a staining solution for 72 h at room temperature. The staining solution contained 5 g each of black tea (Teekanne GmbH, Düsseldorf, Germany), dark soy sauce (Premium dark soy sauce, a Chinese brand), espresso (Nescafe type espresso, Nestle AG, Frankfurt am Main, Germany), and Maggi sauce (Maggi Würze, Maggi GmbH, Germany). Each ingredient was added and mixed to 100 ml of water at 95 °C and left to cool down at room temperature for 20 min before using [26]. The solution was filtered using a cellulose filter made of natural fibers (Teefilter, size 3, Profissimo, dm-drogerie markt, Karlsruhe, Germany) and its pH was set at 5.5 at 23 °C using an electronic pH meter (WTW bench pH/mV meters Routine meter pH 526, Sigma-Aldrich Chemie GmbH, Taufkirchen, Germany). After taking out the samples from the staining solution, they were rinsed with water and blot dried with a paper towel to remove loose extrinsic colorants.

### Treatment procedures

Prepared samples were randomly assigned to five groups (n = 10). The experimental groups differ concerning treatment agents. Samples in the first group were treated with a mixture of 6.25 wt% HAP, 5 ml P11-4 peptide (Curodont Repair, Credentis AG, Windisch, Switzerland), and lactic acid, using NaOCl 3% as pre-treatment. Second, third and fourth groups were treated with 6.25 wt% HAP, 5 ml P11-4 peptide (Curodont Repair, Credentis AG, Windisch, Switzerland), and NaOCl 3%, respectively. The fifth group served as the control group, in which only water was applied (Table 1).

**Table 1:** Treatment, pre-treatment agents, and color measurement devices used in each experiment group

Group name	Treatment agent			Pre-treatment
	Protein	HAP	Water	NaOCl (3%)
gp1-HPN	✓	✓		✓
gp2-H		✓		
gp3-P	✓			
gp4-N				✓
gp5-W			✓	

Each sample in the first group was initially rinsed with water and then NaOCl (3%) was continuously applied on its surface, using a micro-brush (BRAND Micro-brush, Grafton, USA). After 30 s of exposure time, the sample was rinsed thoroughly with water, and then it was placed within the sample holder. Subsequently, the baseline color was measured. To prepare the protein solution, Curodont repair crystals were dissolved and rehydrated with 0.05 ml of sterile water and mixed with 10 ml of 6.25% HAP,^2^ using a Vortex mixer (neoLab, Heidelberg, Germany). To Trigger the polymerization of the P11-4 peptide, the lactic acid was mixed with the suspension using an Eppendorf pipette (Eppendorf Vertrieb Deutschland GmbH, Wesseling- Berzdorf, Germany) and the pH of the suspension was optimized (slightly acidic). The suspension was directly applied to the sample surface for a continuous 30 s, using a fine disposable micro brush. The sample was rinsed with water and subsequently, a soft absorbent paper towel was used to blot dry it (half-dry). After 90 s of air exposure, the color was measured again.

In the second to fifth groups, each sample was initially rinsed with water and blot dried with a soft paper towel, and its baseline color was measured. Then, the respective treatment agent (water in gp5-W) was applied continuously on the sample surface for 30 s. Finally, the sample was rinsed thoroughly with water and after 90 s of air exposure time, its color was measured again.

### Color measurement

Sample color was determined using the Ocean Optics spectrophotometer (USB4000-VIS–NIR-ES, Ostfildern, Germany). To prevent errors associated with color measurement, the measurement area was darkened as per the recommendation of the spectrophotometer’s manufacturer.

In this study, the color changes were evaluated according to the CIELAB color scale. In this system, L*indicates lightness/brightness, a* represents redness-greenness, and b* is yellowness-blueness coordinate. Differences in L*, a*, and b* between baseline and after each treatment were expressed as ΔL*, Δa*, and Δb*, respectively. Overall color difference (ΔE) was calculated using the following equation: $\Delta E = \sqrt {\left( {\Delta L^{*} } \right)^{2} + \left( {\Delta a^{*} } \right)^{2} + \left( {\Delta b^{*} } \right)^{2} }$. Furthermore, for a more comprehensive assessment, the customized CIELAB-based whiteness index introduced by Pérez *et* al. [27], was used in the first group (gp1-HPN).

### Statistical analysis

The mean and standard deviation of overall color changes were calculated and statistically analyzed using IBM SPSS statistics (version 18.0). Data were tested for normality using the Shapiro Wilk test; the level of significance for all tests was set at 0.05.

To compare the impact of the treatment agents/approaches on the overall degree of color changes (ΔE), ANOVA tests with post-hoc Tukey’s Honestly Significant Difference (HSD) were used. To compute the statistical power of the test, the G*Power tool (version 3.1) was used.

### SEM evaluation

Two bovine incisors were randomly selected, polished, and stained for 72 h at 24 °C. The labial tooth surfaces were polished using a 1200-grit abrasive paper under water cooling. Then, small blocks (5 mm × 5 mm × 1 mm) were prepared from the labial enamel surface from comparable regions (middle one-third) of each tooth [28]. For a simplified handling, enamel blocks were glued onto glass slides, and samples were assigned into two groups (n = 1).

The first sample (SEM1-w-NaOCl) received a pre-treatment with 3% NaOCl for 30 s and then rinsed with water. Subsequently, the sample was treated with the suspension used in gp1-HPN, which was a mixture of 6.25 wt% HAP and 5 ml P11-4 peptide.

The second sample (SEM2-wo-NaOCl) was treated with the same suspension without receiving any pre-treatment. Both samples were rinsed with water after treatment and subsequently, a soft absorbent paper towel was used to dry them. After 24 h, samples were sputter-coated with an ultrathin layer of gold–palladium alloy (SC7620, Polaron, QuorumTechnologies, Kent, UK). SEM images were taken at two magnifications (× 5000 and × 10,000) using a scanning electron microscope (SEM, ZEISS Supra 55vp, Zeiss, Oberkochen, Germany).

## Results

### Effect of treatment procedure on color changes

**Fig. 1:**
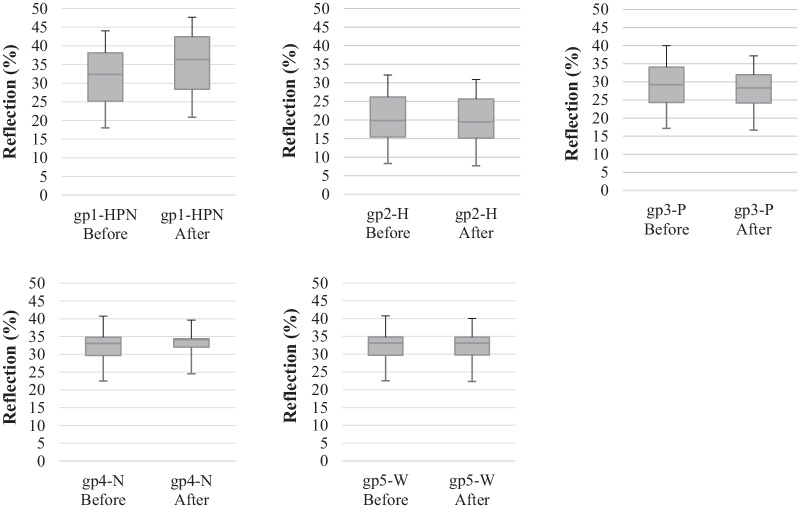
Box-and-whisker plot of the average reflections of each group before treatment (i.e., baseline) and after treatment with the respective treatment agent

The box-and-whisker plots in Fig. 1 compare the range of reflections over the wavelengths from 360 to 750 nm for each of the experimental groups before treatment (i.e., baseline) and after treatment. gp1-HPN presented considerable differences between the baseline and after the treatment. The median of the reflections in this group increased from 32.4 in baseline, to 36.3 after treatment with the HAP-peptide suspension. In contrast, the application of HAP in the gp2-H did not result in a notable deviation from the baseline reflections. The median of this group decreased slightly from 19.8 to 19.5 after the treatment. Treatment with peptide reduced the median value of the gp3-P from 29.2 to 28.4, whereas application of NaOCl in gp4-N increased the median value from 33.1 to 34.1. It was not surprising that the reflections of the samples in the control group (gp5-W) did not remarkably change after the treatment with water.

To assess the overall color differences between the treatment procedures, we compared the mean ΔE values of the groups. Table 2 presents relevant descriptive statistics, including mean, standard deviation, 95% confidence intervals, minimum and maximum for the ΔE values of each treatment group, as well as of all groups together. It can be seen that gp1-HPN, with the mean ΔE of 4.64, presented the most pronounced overall color change compared to the other groups. In contrast, gp5-W presented the lowest mean with 0.99. It is noteworthy that gp1-HPN also presented the largest standard deviation (4.25). As indicated in the table, the delta between the minimum (0.32) and maximum (13.22) of this group was the most significant.

**Table 2:** Descriptive statistics of ΔE values of each group and all samples together

Group	Paired differences					N	Min	Max
	Mean	Std. Deviation	Std. Error Mean	95% Confidence Interval of the difference				
				Lower	Upper			
gp1-HPN	4.64	4.25	1.34	1.59	7.68	10	0.32	13.22
gp2-H	1.27	1.32	0.42	0.32	2.21	10	0.11	4.14
gp3-P	2.33	1.71	0.54	1.11	3.55	10	0.22	5.50
gp4-N	3.90	2.78	0.88	1.91	5.89	10	1.36	10.80
gp5-W	0.99	1.17	0.37	0.15	1.83	10	0.30	4.20
Total	2.62	2.82	0.40	1.82	3.43	50	0.11	13.22

Next to water, the application of HAP showed the weakest color change ($\Delta {\text{E}} = {1}.{27} \pm {1}.{32}$). With $\Delta {\text{E}}$ of 2.33 ± 1.71, treatment with peptide also presented a relatively slight color change. Application of NaOCl which is supposed to be used as pre-treatment, resulted in a notable overall color change ($\Delta {\text{E}} = 3.{9}0 \pm {2}.{78}$).

Results of the statistical test show that the significance value between the groups was 0.007 (i.e., *p* = 0.007). This rejected the null hypothesis and confirmed that there was a statistically significant difference between the mean ΔE values of the groups (Table 3). It is noteworthy that the statistical power of the test was found to be significantly high (greater than 90%).

**Table 3:** Results of the one-way ANOVA test for the ΔE values of the groups

	Sum of squares	*df*	Mean square	F	Sig
Between groups	102.75	4	25.69	4.03	0.007
Within groups	286.92	45	6.38		
Total	389.66	49			

Table 4 provides the results of the post-hoc test. Although gp1-HPN showed more obvious overall color change than gp3-P and gp4-N, the differences between these groups were not statistically significant. In contrast, gp1-HPN presented significantly stronger overall color change compared to gp2-H (*p* = 0.04) and gp5-W (*p* = 0.02). It was observed that the differences between gp5-W with gp2-H, gp3-P and gp4-N were not statistically significant.

**Table 4:** Results of the Tukey’s HSD test for the ΔE values of the groups

(I) Groups	(J) Groups	Mean difference (I–J)	Std. Error	Sig	95% Confidence Interval	
					Lower bound	Upper bound
gp1-HPN	gp3-P	2.31	1.13	0.26	− 0.90	5.52
	gp2-H	3.37	1.13	0.04	0.16	6.58
	gp4-N	0.74	1.13	0.97	− 2.47	3.95
	gp5-W	3.65	1.13	0.02	0.44	6.85
gp2-H	gp1-HPN	− 3.37	1.13	0.04	− 6.58	− 0.16
	gp3-P	− 1.06	1.13	0.88	− 4.27	2.15
	NaOCl	− 2.63	1.13	0.15	− 5.84	0.58
	gp5-W	0.28	1.13	1.00	− 2.93	3.49
gp3-P	gp1-HPN	− 2.3	1.13	0.26	− 5.52	0.90
	gp2-H	1.06	1.13	0.88	− 2.15	4.27
	NaOCl	− 1.57	1.13	0.64	− 4.78	1.64
	gp5-W	1.34	1.13	0.76	− 1.87	4.55
gp4-N	gp1-HPN	− 0.74	1.13	0.97	− 3.95	2.47
	gp3-P	1.57	1.13	0.64	− 1.64	4.78
	gp2-H	2.63	1.13	0.15	− 0.58	5.84
	gp5-W	2.91	1.13	0.09	− 0.30	6.12
gp5-W	gp1-HPN	− 3.67	1.13	0.02	− 6.85	− 0.44
	gp3-P	− 1.34	1.13	0.76	− 4.55	1.87
	gp2-H	− 0.28	1.13	1.00	− 3.49	2.93
	gp4-N	− 2.91	1.13	0.09	− 6.12	0.30

Suffice to state that although neither HAP nor peptide nor NaOCl resulted in significantly different overall color change compared to the control group, mixing HAP with peptide and using NaOCl as pre-treatment presented significantly stronger overall color change than the control group (gp5-W).

**Fig. 2:**
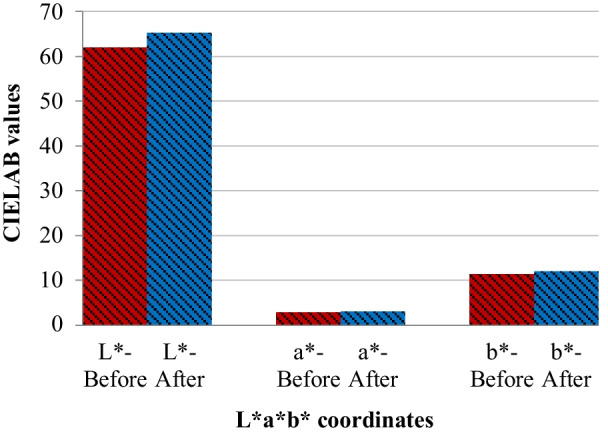
The mean CIELAB values before and after the treatment with the main suspension

It was recognized that the treatment procedure in gp1-HPN had a significant effect on the overall color of the samples. To provide a more detailed assessment, we compared the underlying CIELAB values before and after treatment. As presented in Fig. 2, the treatment procedure in this group increased all three CIELAB values. In absolute terms, L^*^ experienced the strongest growth ($\Delta {\text{L}}^{*} = {3}.{22}$), followed by b^*^ ($\Delta {\text{b}}^{*} = 0.{67}$). The red-green factor (a^*^) increased from 2.89 in the baseline to 3.11 after the treatment with HAP-peptide ($\Delta {\text{a}}^{*} = { }0.{22}$) (Table 5). The mean $\Delta {\text{WI}}_{{\text{D}}}$ of this group was equal to + 1.05.

**Table 5:** Mean values of $\Delta {\text{L}}^{*}$, $\Delta {\text{a}}^{*}$ and $\Delta {\text{b}}^{*}$ the associated color perception for the gp1-HPN group

	$\Delta {\text{L}}^{*}$	$\Delta {\text{a}}^{*}$	$\Delta {\text{b}}^{*}$
Mean value	+ 3.22	+ 0.22	+ 0.67
Color perception	Lighter	Redder	Yellower

### SEM evaluation

**Fig. 3:**
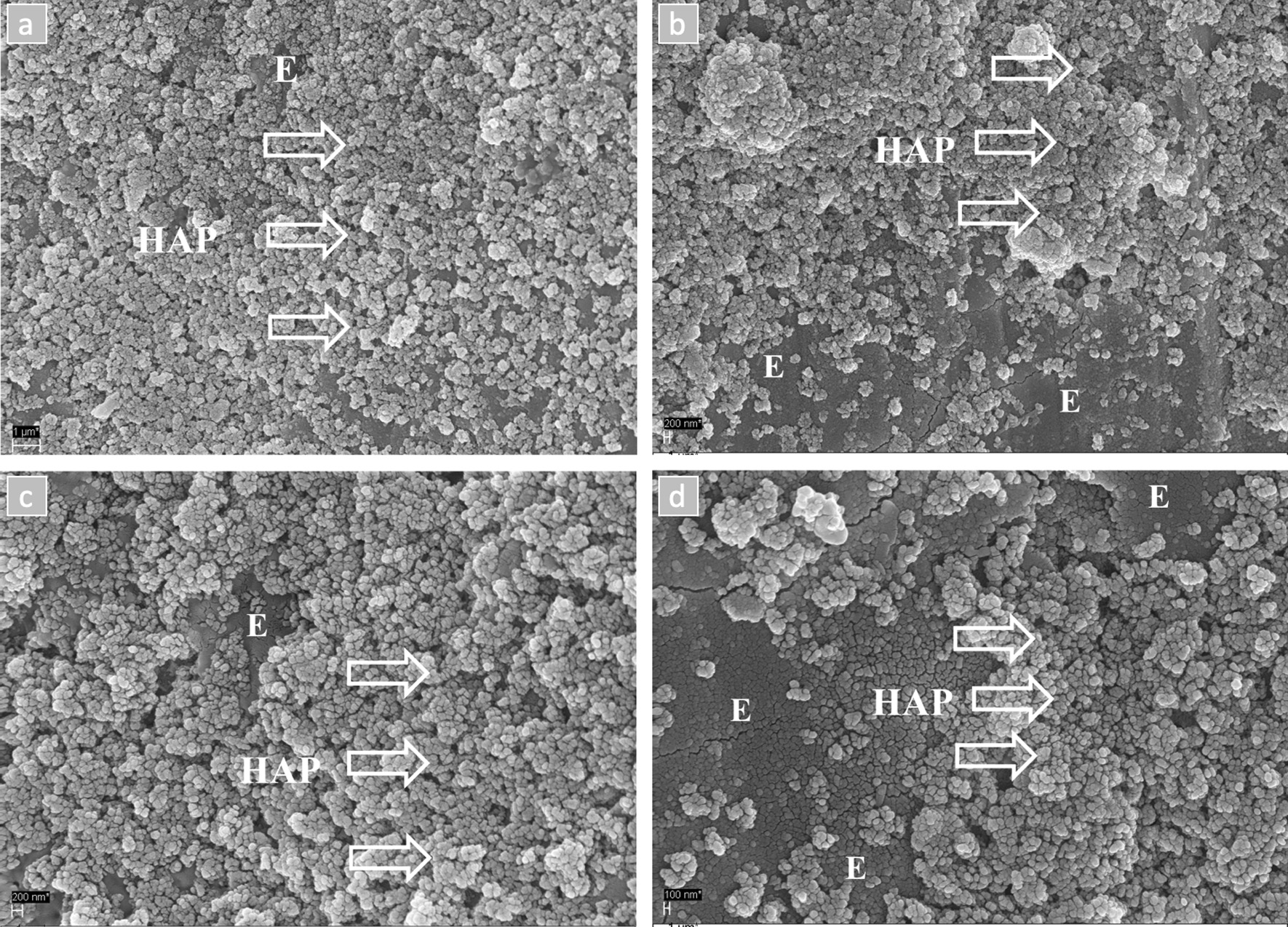
SEM images illustrating the bovine enamel surface coated with the adhered HAP particles with and without pre-treatment with NaOCl at × 5000 (**a**–**b**) and × 10,000 magnifications (**c**–**d**). The first sample (SEM1-w-NaOCl) received a pre-treatment with 3% NaOCl, rinsed with water and then were treated with the peptide-HAP (6.25 wt %) suspension resulted in a more pronounced and denser coverage of the enamel surface (E) with HAP particles (arrows) (**a**, **c**). The second sample (SEM2-wo-NaOCl) was treated with the same suspension without receiving any pre-treatment with NaOCl, which showed a layer with many HAP particles and HAP agglomerates (arrows) covering a considerable area of the enamel surface (E) (**b**, **d**)

As illustrated in Fig. 3, SEM1-w-NaOCl revealed a continuous or seamless layer of HAP particles covering the whole enamel surface. This was noticeably more pronounced than the coverage in SEM2-wo-NaOCl.

## Discussion

Due to its suitable characteristics and inherent biocompatibility, the P11-4 peptide was used in this study to promote and stabilize the whitening effect of HAP on the samples. In fact, the formation of the 3D scaffold may allow for a stronger adhesion of HAP particles on the tooth surface. To expose a maximum amount of binding sites of the enamel surface to the treatment suspension (containing peptide and HAP), this study aimed at discarding the organic components of the smear layer. To this end, NaOCl was used as a pre-treatment agent. It is important to note that NaOCl can have a bleaching effect. However, for a significant bleaching effect, it would be necessary to use a high concentration of NaOCl for a considerably long application time. For instance, NaOCl 5% should be continuously applied for at least 25 min to have a detectable bleaching effect [29]. Therefore, to avoid any undesirable bleaching effect, we used NaOCl with a concentration of 3% for a very short period (30 s). Our results confirmed that the application of NaOCl 3% did not have any significant impact on the overall color changes. It is also noteworthy that in the gp1-HPN group in which NaOCl was applied as pre-treatment, the baseline color of the samples was measured after the pre-treatment to ensure that NaOCl did not interfere in the overall color changes ($\Delta {\text{E}}$) of this group.

Our findings showed that similar to NaOCl, separate applications of HAP and P11-4 did not exert an acceptable impact on the overall color changes. In contrast, treatment of the samples with a mixture of P11-4 and HAP after pre-treatment with NaOCl resulted in significant overall color changes. Therefore, the underlying hypothesis of our study can be accepted. It was observed that the ΔE mean value of the gp1-HPN group was higher than 4, indicating that the overall color changes in this group were perceptible. Increasing the L* value in this group can be explained by increasing the reflection of light on the HAP layer and decreasing light transmission through HAP on the enamel (Table 5). In addition, our results confirmed that the application of HAP-Peptide also increased the mean customized whiteness index (WI_D_) of the gp1-HPN group. According to Pérez *et* al. [30], WI_D_ has a high correlation with visual perception. The analysis also showed that only in the gp1-HPN group which received the pre-treatment, the mean reflection increased significantly (Fig. 1). This is in line with our hypothesis that the presence of a barrier on the surface may have a negative impact on the adhesion of HAP particles.

The SEM investigation also confirmed a more pronounced and denser coverage of adhered HAP particles on the enamel surface after using NaOCl as pre-treatment.

It is worth highlighting that using NaOCl may cause some adverse side effects such as mucosa ulceration, damages to the eye and skin, as well as allergic reactions [25, 31, 32]. Due to its aggressiveness, before any treatment with NaOCl, the teeth should be isolated with a rubber dam to protect the soft tissue. Therefore, patients are not allowed to use this agent without the supervision of a dentist or a professional.

P11-4 peptide can form an organic 3D matrix. This matrix is highly affine to Calcium and Phosphate ions. The peptide contains anionically charged phosphoserine residues which may attract Calcium ions, providing a heterogeneous nucleator for HAP crystal formation. Furthermore, these fibers bind to the already existing Calcium ions of the HAP of the enamel.

Based on our observation, generating a new HAP layer on the enamel surface made the surface smoother. This leads to increased brightness and whiteness, which is due to an increase in diffuse reflection of light. This effect is not permanent, but the treatment can be repeated without undesirable effects, subject to finding a safer alternative agent as pre-treatment. This can be further investigated by future studies.

Acid etching is a commonly used method to cause scratches and increase surface porosity [33]. In several studies that dealt with P11-4 to treat initial caries lesions, an acid was applied to open the micropores before the application of the peptide [34, 35]. This was driven by the fact that remineralization of a more porous enamel surface is much easier [36]. In the present study, a pre-treatment with an etching solution was not performed because it would have increased the porosity and roughness, which were not our desire. In fact, our aim was to make the teeth appear whiter without causing any enamel damages. This would represent another advantage of the proposed approach compared to commonly available bleaching methods which may increase enamel roughness.

The pH of the suspension used in the gp1-HPN group was set to approximately 6.6 (slightly acidic). At this pH, the viscosity decreases and as a result the peptide switches in the fluid nematic state (viscoelastic fluid). Moreover, this slightly acidic suspension is too weak to cause any damages to the robust dental tissue during the treatment.

There are some minor differences in chemical and physical characteristics between bovine and human enamel [37–39]. For instance, the size of bovine enamel crystals is slightly bigger than that of human enamel crystals. However, since the differences are tolerable, bovine enamel has been frequently used as the most common alternative for human enamel (e.g., [40, 41]). It is also noteworthy that extracted bovine incisors are more accessible than human ones [42].

The enamel surface was polished to minimize individual enamel surface variations, simulate some possible conditions that may happen in the human oral cavity (e.g., erosion and abrasion), and standardize the measurement area. Furthermore, we polished the enamel surface to remove the aprismatic layer which contains more minerals than the enamel subsurface. This layer is less permeable to treatment agents and acidic solutions than the underlying enamel [43]. Surface grinding also facilitated removing exogenous discolorations. On the other hand, the native tooth surface is slightly curved and shines strongly. As a result, the proportion of specular light reflection is high. The curvature makes the light’s reflection direction unpredictable. Grinding the enamel surface flat also minimized the unreliability caused by strong light reflection from the curved surface.

One of the advantages of the proposed suspension is the treatment of white spot lesions which may happen due to poor oral hygiene and plaque, bacteria, and acid accumulation on teeth. Since white spot lesions are a common adverse effect of fixed orthodontic appliances, this mixture can be beneficial for orthodontics patients [44]. Future studies are required to investigate the application of the proposed suspension during and after orthodontics treatments.

While no major adverse side-effects have been reported so far for P11-4, there might be some concerns about the potential health risks associated with long-term oral exposure to Calcium phosphate nanoparticles [45]. However, in case during tooth brushing these particles are accidentally swallowed, the highly acidic pH of the stomach helps dissolve them completely [46]. In fact, the potential health risk is very limited, especially at low exposure levels [2]. Therefore, as also discussed in [19], P11-4-HAP is a safe and painless tooth whitening agent, which can be used as a mouthwash or a dental foam on a daily basis without the dentist's supervision.

The application of P11-4 improves the enamel microhardness [47]. The P11-4-HAP suspension may also be effective in the treatment of hypersensitive teeth. In contrast, Hydrogen peroxide reduces the enamel microhardness, increases its roughness, and may cause tooth sensitivity. Adding HAP-containing suspension to peroxide-based bleaching agents may reduce the respective adverse effects of the bleaching agents [20, 48, 49]. However, the application of HAP during or after the bleaching process does not interfere with the bleaching effect [50].

One of the critical challenges in pediatric dentistry is dealing with hypersensitive, discolored, and chalky teeth, which occur in molar hypomineralization. Regular use of the proposed suspension could be beneficial in the treatment of the respective involved teeth. This can be further explored by future works in this context.

## Conclusions

According to the present study, using NaOCl as a pre-treatment can improve the adherence of the HAP-peptide layer on the enamel surface and enhance the coverage of the surface by HAP particles, resulting in a stronger whitening result.

## Data Availability

The experimental data used and/or analyzed during the current study are available from the corresponding author on reasonable request.

## Abbreviations

HAP: Hydroxyapatite
NaOCl: Sodium hypochlorite
WI_D_: Whiteness Index in Dentistry

## Footnotes

1 Correspond to polishing particles of an average size of 16 μm and 6.5 μm, respectively.

2 The initial concentration was 25%.
